# Prevalence and correlates of suicidal ideation in Korean firefighters: a nationwide study

**DOI:** 10.1186/s12888-019-2388-9

**Published:** 2019-12-30

**Authors:** Heyeon Park, Johanna Inhyang Kim, Beomjun Min, Sohee Oh, Jeong-Hyun Kim

**Affiliations:** 1 Department of Public Health Medical Services, Seoul National University Bundang Hospital, 82 Gumi-ro 173 Beon-gil, Bundang-gu, Seongnam-si, Gyeonggi-do 13620 South Korea; 2 Department of Psychiatry, Hanyang University Medical Center , 222-1, Wangsimni-ro Seongdong-gu, Seoul, South Korea; 3 Department of Biostatistics, Seoul Metropolitan Government Seoul National University Boramae Medical Center, Boramae-ro 5-gil, Dongjak-gu, Seoul, South Korea; 4 Mental Health and Behavioral Medicine Services for Clinical Departments, Seoul National University Bundang Hospital, 82 Gumi-ro 173 Beon-gil, Bundang-gu, Seongnam-si, Gyeonggi-do 13620 South Korea; 5 Department of Psychiatry, Seoul National University Bundang Hospital, 82 Gumi-ro 173 Beon-gil, Bundang-gu, Seongnam-si, Gyeonggi-do 13620 South Korea

**Keywords:** Firefighters, Suicidal ideation, Prevalence, Occupational stress, Emotional labor

## Abstract

**Background:**

It is generally known that firefighters are at increased risk of suicide. However, the prevalence and correlates of suicidal ideation in firefighters have not been thoroughly described to date. The aim of this study was to measure the 1-year prevalence of suicidal ideation in firefighters and to investigate the correlates of past-year suicidal ideation among the demographic, occupational and clinical characteristics.

**Method:**

A web-based survey was conducted using a self-reported questionnaire. A total of 45,698 Korean firefighters were included for analysis. The prevalence of suicidal ideation in the past year was calculated and its correlates were elucidated using a multivariable logistic regression analysis.

**Results:**

The 1-year prevalence of suicidal ideation was 10.66% in Korean firefighters. Recent traumatic experience, high levels of occupational stress from physical work environment and emotional labor, as well as current duty of officer were significant correlates of suicidal ideation in the previous year, even after controlling for the effects of PTSD and depressive symptoms. With respect to demographic factors, female gender and marital status of divorced/separated/widowed were identified to be associated with suicidal ideation in the previous year among firefighters.

**Conclusions:**

The 1-year prevalence of suicidal ideation was high in Korean firefighters and was associated with various occupational factors as well as psychiatric symptoms. Early detection and management of these risk factors could reduce the risk of suicidal ideation in firefighters.

## Background

Suicide and associated behaviors are major public mental health issues around the world. Suicide is one of the leading causes of death worldwide for adults [1]. South Korea, in particular, is a country with a markedly high rate of suicide for the past several years. According to the World Health Organization, the suicide rate in South Korea was 26.9 per 100,000 persons, ranking 4th highest in the world [2, 3]. One way to prevent suicide is to identify the risk factors associated with increased suicidal behaviors, including ideation, plan, and attempt, since they have been found to be predictors for completed suicide [4–6]. Especially, suicidal ideation is strongly associated with a variety of psychological difficulties and subsequent suicide attempts [7, 8]. Therefore, factual survey and investigation of suicidal ideation in various populations are needed to prevent suicide.

One occupation group that particularly deserves such attention is firefighters because they frequently experience traumatic events that may increase suicidal behaviors [9, 10]. It was recently reported that the number of firefighters who died from suicide was greater than the number who died in the line of duty in United States [11]. In South Korea, there were also more suicides than on-the-work deaths in firefighters during the last decade, according to an informal report from the National Fire Agency. However, the rate of suicide and suicidal behaviors among firefighters are markedly less investigated compared with other groups of first responders, such as police officers [12]. One previous study has tried to determine the career prevalence of suicidal behaviors, including suicidal ideation, plans, and attempts by analyzing data obtained from a sample of 1027 current and retired firefighters in the United States (US). This study reported very high rate of suicidal ideation, plan, and attempt in US firefighters compared with the general population [13]. However, this study included an integrated population of firefighters, ranging from volunteers to retired firefighters in the US; therefore, it may be necessary to conduct research on firefighters who are currently working as professional in other countries. Moreover, this previous study employed a retrospective survey on whole career of participants, so it is highly possible that the participants’ perspective on their suicidal behaviors may have been skewed. To investigate suicidal ideation related to firefighters’ work and its various correlates, research on data of recent suicidal ideation is needed.

Psychiatric symptoms, such as depression, posttraumatic stress disorder (PTSD) symptoms [14, 15], and sleep disturbances [16], were revealed to be major risk factors associated with suicidal behaviors in firefighters. In addition, lower rank, fewer years of firefighter service, active duty military status, history of professionally responding to suicidal attempt or death cases, and membership in an all-volunteer department were reported as occupational correlates of suicidal ideation among firefighters [13]. However, it is still uncertain whether and how work-related stress in their occupational environment impacts suicidal ideation in firefighters, despite attempts to identify the predictors for suicidal behaviors in this population. Previous epidemiological studies showed a potential association between occupational stress and suicidal ideation in emergency workers. Specifically, job-related emotional exhaustion and bullying at work were associated with suicidal ideation in Norwegian ambulance personnel [17]. Also, having been harassed at work was associated with suicidal ideation among physicians in academic medicine [18]. Considering these findings, occupational stress might be associated with suicidal ideation in such population.

Several occupational stressors unique to firefighters are necessary to be investigated. First, traumatic experience while on duty might be the highest risk factor related to suicidal ideation. Traumatic experiences in firefighters have been widely acknowledged as a major risk factor for various mental disorders. The severity of PTSD symptoms and traumatic experience were positively related with the risk of lifetime suicidal ideation and attempts [14]. However, it remains unknown whether and how a recent exposure to traumatic event impacts suicidal ideation in firefighters. Second, occupational stress from risky or hostile workplace environment might also be a risk factor for suicidal ideation in firefighters [19, 20]. In particular, firefighters routinely suffer from harsh working condition, exposed to physical danger while on duty, outdoor work regardless of weather, handling of heavy equipment, and irregular work hours. Investigations are needed to determine whether occupational stress related to the characteristics of the physical work condition is one of the correlates of suicidal ideation in firefighters. In addition, emotional labor might also be one of the occupational stressors. Emotional labor is the process of controlling feelings in compliance with the organizational demands and occupational duties [21–23]. Firefighters have to hide their own emotions when they face sickness, death, suicide, and violent accidents. Moreover, it has been found that firefighters usually suffer from emotional labor similar to those in customer-service industry due to the aggressive and/or picky nature of civil petitioners in Korea [24]. Considering the previous findings that emotional labor has a negative impact on mental health among workers [25–29], it is needed to investigate whether emotional labor is associated with suicidal ideation in firefighters.

In this study, we conducted a nationwide survey on past-year suicidal ideation and its correlates among 45,698 firefighters in South Korea. The purpose of this study was twofold: 1) to determine the 1-year prevalence of suicidal ideation in firefighters and 2) to investigate whether and how demographic characteristics (age, sex, marital status, and religion), occupational factors (current duty, traumatic experience in the previous year, stress from physical work condition, and emotional damage from emotional labor), and clinical symptoms (PTSD and depression) are correlated with past-year suicidal ideation in this population.

## Methods

### Participants

This nationwide cross-sectional study was conducted between February 2018 and March 2018 via a self-reported online survey among Korean firefighters. A total of 45,719 firefighters in South Korea participated in the survey and completed the self-reported questionnaire, including their suicidal ideation in the past year, as well as demographic characteristics and occupational factors, such as traumatic experience in the past year, occupational stress, emotional labor, and clinical factors, including PTSD and depression symptoms. Among the total, 21 were excluded from the final analyses due to coding error during the survey. Thus, the final analysis included a total of 45,698 firefighters. Survey respondents were apprised of the anonymous and voluntary nature of the self-reported online survey.

### Measures

#### Demographic and occupational characteristics

The demographic and occupational characteristics were obtained using a self-reported questionnaire. Demographic characteristics included age, sex (male or female), marital status (married, never married, or divorced/separated/widowed), and religion (yes or no). Occupational characteristics included length of work (years) and current duty. The roles of firefighters include fire suppression, special investigation of the cause of fire, paramedics providing emergency medical care, rescuing people who are trapped or in medical emergencies, training other firefighters, and others [30]. For analysis, the roles were categorized into the following: fire suppression, emergency medical services (EMS: includes paramedics and rescue), and officers (including administrators, special investigators, trainers of firefighters, and communicational and informational system operators).

#### Suicidal ideation in the past year

Suicidal ideation in the previous year was assessed using an item of the Suicidal Behavior Questionnaire-Revised (SBQ-R) [31]. The SBQ-R is a brief self-reported questionnaire to inquire about the various aspects of suicidal behaviors. Item 1 explores whether the respondents have ever thought about or attempted suicide in his/her lifetime; item 2 evaluates how often the respondents have thought about suicide over the past 12 months; item 3 assesses the threats of suicide attempts; and item 4 explores the self-reported likelihood of suicidal behaviors in the future. In this study, we used item 2 to assess past-year suicidal ideation in participants. Item 2 reads: “How often have you thought about killing yourself in the past year?” Participants responded to this item on a 5-point Likert scale: 1-never; 2-rarely (1 time); 3-sometimes (2 times); 4-often (3–4 times), and 5-very often (5 or more times). A score of greater than 2 on item 2 was indicated as having suicidal ideation more than once in the past year. According to the validation study for the SBQ-R, item 2 had the largest effect size followed by item 1 for differentiating between suicidal-risk and non-suicidal participants in both clinical and nonclinical adult sample population [31]. The correlation coefficient between SBQ-R item 2 and PHQ-9 item 9, which inquires about the frequency of suicidal thought over the last 2 weeks, was *r* = 0.499 (*p* <  0.001). Additionally, the correlation coefficient between SBQ-R item 2 and the total score of PHQ-9 was *r* = 0.479 (*p* <  0.001).

#### The presence of recent exposure to traumatic events

Exposure to traumatic events during the previous year was identified by using the self-reported measure – developed by Beaton et al. – which assessed duty-related incident stressors [32]. Twenty-two items were selected among the original 33 incident stressors based on the previous result of rating the stressfulness of the 33 stressors [32]. We excluded two stressors related to gunshots due to the generally low rate of gunshot incidents due to strict gun control laws in South Korea. In South Korea, only government-authorized personnel can own or carry guns. Gun culture is notably absent outside of the military, and gun ownership and death rank among the lowest in the world [33]. ‘Witness duty-related death of co-worker’ and ‘co-worker firefighter fire fatality (not witnessed)’ were changed to ‘witness duty-related death or suicide of co-worker’ and ‘co-worker death or suicide (not witnessed), respectively. Finally, three additional stressors, ‘remove the body of a suicide victim’, ‘remove a severely decayed corpse’, and ‘involved in a safety accident that received public spotlight’, which were reported to be frequently encountered and associated with high level of stress in Korean firefighters, were added (Additional file 1: Table S1). Participants were asked whether they were exposed to each stressor in the previous year. More than one exposure to traumatic events in the previous year was regarded as having recent exposure to traumatic events.

#### Occupational stress from physical work environment

Occupational stress from physical work environment was measured using the subscale, ‘Difficult Physical Environment’ of the Korean Occupational Stress Scale (KOSS) [34], which was developed and validated using a nationwide epidemiological study to estimate job stress in Korean employees. The KOSS was based on the most commonly used job stress questionnaires, such as the Job Content Questionnaire [35], National Institute of Occupational Safety and Health job stress questionnaire [36], and Occupational Stress Index [37]. The KOSS has eight subscales (Difficult Physical Environment, High Job Demand, Insufficient Job control, Inadequate Social Support, Job Insecurity, Organizational Injustice, Lack of Reward, and Discomfort in Occupational Climate). The “Difficult Physical Environment’ of the KOSS has three items, each of which was rated on a 4-point Likert scale (1: strongly disagree to 4: strongly agree); higher scores represented higher levels of occupational stress from physical environment. Additional file 2: Table S2 illustrates each item on the “Difficult Physical Environment” subscale of the KOSS. The internal consistency of “Difficult Physical Environment” of KOSS based on the presented sample was *α* = 0.464.

#### Emotional labor

Emotional labor was assessed by the Korean Emotional Labor Scale (KELS) [38]. The KELS was developed and validated in a nationwide study to measure emotional labor in Korean employees by the Korean Occupational Safety & Health Agency. The KELS was based on the previous studies [21–23, 39], the most commonly used emotional labor scales [6, 40, 41], and a focused group interview. The KELS has five subscales which are Effort to Control Emotion, Organizational Monitoring System, Demands of Emotional Labor, Emotional Damage, and Organizational Support System. Each item of the KELS was rated on a 4-point Likert scale, from 1 (not at all) to 4 (very much), and higher scores indicated higher levels of stress from emotional labor. The current study included only the subscale, “Emotional Damage,” which measures the severity of emotional hurt due to emotional labor. We regarded the subscale score as proxies for emotional labor in firefighters, because “Emotional Damage” is a factor that explained the most variance of the KELS in the results of a factor analysis in a previous study that developed the scale [38]. Additional file 2: Table S2 shows each item in the “Emotional Damage” subscale of the KELS. The internal consistency of “Emotional Damage” of KELS based on the presented sample was *α* = 0.947.

#### PTSD symptoms

PTSD symptoms were assessed with the Korean version of PTSD Checklist-for the fifth edition of the Diagnostic and Statistical Manual of Mental Disorders (PCL-5) [42]. The PCL-5 is a 20-item self-reported measure evaluating the degree to which an individual has been bothered in the past month by DSM-5 (Diagnostic and Statistical Manual of Mental Disorders, 5th edition) PTSD symptoms [43]: intrusions, avoidance, negative alteration in cognition and mood, and alterations in arousal and reactivity. We instructed the participants to choose and describe the most traumatic event from a list of traumatic events and fill out the PCL-5 with this event in mind. Each item was measured on a 5-point Likert scale (0: not at all to 4: extremely). Higher scores represented higher severity of PTSD symptoms. Items 1–5 correlated with symptoms within Cluster B (intrusions); items 6–7 with Cluster C (avoidance); items 8–14 with Cluster D (negative alteration in cognition and mood); and items 15–20 with Cluster E (alterations in arousal and reactivity). The internal consistency of PCL = 5 based on the presented sample was *α* = 0.961. Participants were considered to be experiencing the symptom when recording a score of 2 or higher (moderately to extremely) in each item. According to an algorithm-derived PTSD diagnosis method, we defined probable PTSD as having the required number of symptoms in each cluster of the DSM-5 criteria: 1 B item, 1 C item, 2 D items, and 2 E items.

#### Depression symptoms

Depression symptoms were assessed using the Korean version of Patient Health Quetionnaire-9 (PHQ-9) [44, 45]. Respondents rated 9 items, based on the DSM-IV criteria of major depressive disorder, measured on a 4-point Likert scale (0: not at all to 3: nearly every day) based on their experiences during the past 2 weeks. The PHQ-9 total score ranged from 0 to 27; higher scores indicated a greater severity of depressive symptoms. The internal consistency of PHQ-9 based on the presented sample was *α* = 0.905. The total score of over 15 was defined as probable depression [45].

### Statistical analysis

Descriptive statistics were used to analyze the demographic, occupational, and clinical characteristics of participants, as well as to calculate the 1-year prevalence of suicidal ideation. Chi-square test and *t-*test were used to examine whether there were differences in the demographic, occupational, and clinical characteristics between firefighters with and without suicidal ideation in the past year. Multivariable logistic regression analysis was used to examine the demographic (age, sex, marital status, and religion), occupational (current duty, recent traumatic experience, occupational stress, and emotional labor), and clinical (probable PTSD and depression) characteristics as correlates of suicidal ideation in the previous year. The dependent variable was suicidal ideation in the past year. In the logistic regression analysis, 143 participants with missing data were excluded; thus, a total of 45,555 participants were included in the analysis. The results were shown as the odds ratios (ORs) and 95% confidence intervals (CIs). The data were analyzed using IBM SPSS Statistics ver. 22.0 software (IBM Corp., Chicago, IL, USA). A two-tailed *p*-value < 0.001 was considered statistically significant.

## Results

The mean age of all 45,698 participants was 42.51 years (standard deviation, SD = 9.10), and 92.6% were males. The average length of work was 13.4 years (SD = 9.43). As a current duty, fire suppression, EMS, and officers were 41.57, 32.10, and 26.33%, respectively. In the past year, 62.52% of participants experienced traumatic events. A total of 4871 (10.66%) firefighters had suicidal ideation more than once in the past year. With respect to clinical characteristics, a total of 1202 firefighters (2.63%) were identified as having probable PTSD using PCL-5, and 561 firefighters (1.23%) were identified as having probable depression using PHQ-9.

The results in Table 1 show that there were differences in the demographic, occupational, and clinical characteristics between firefighters with and without suicidal ideation in the past year. The association of suicidal ideation in the previous year with demographic, occupational, and clinical characteristics was investigated by using a multivariable logistic regression model. The results are shown in Table 2. Suicidal ideation in the past year was associated with female gender (OR = 1.484, 95% CI = 1.328–1.657) and with relationship status as divorced/separated/widowed (OR = 1.724, 95% CI = 1.432–2.076). As current duty, officers were more likely to be related to suicidal ideation in the past year (OR = 1.488, 95% CI = 1.366–1.622), compared with fire suppression. The presence of recent trauma (OR = 1.847, 95% CI = 1.709–1.997), higher occupational stress (OR = 1.191, 95% CI = 1.164–1.219), higher emotional labor (OR = 1.095, 95% CI = 1.087–1.103), probable PTSD (OR = 4.008, 95% CI = 3.499–4.591), and probable depression (OR = 8.916, 95% CI = 7.201–11.039) were also significantly associated with suicidal ideation in the past year.

**Table 1:** The demographic, occupational, and clinical characteristics by suicidal ideation in the past year (*N* = 45,698)

	N (%) of M ± SD		T or Chi-square
	Suicidal ideation	No suicidal ideation	
Age	43.01 ± 8.52	42.45 ± 9.16	*t* = 4.31*
Sex			
Male	4344 (89.18)	37,982 (93.03)	*χ2* = 91.42*
Female	527 (10.82)	2845 (6.97)	
Marital status (*N* = 45,606)			
Married	3753 (77.22)	30,813 (75.62)	*χ2* = 98.86*
Never married	929 (19.12)	9195 (22.57)	
Divorced/separated/widowed	178 (3.66)	738 (1.81)	
Religion (*N* = 45,555)			
No	3101 (63.96)	26,532 (65.18)	*χ2* = 2.81
Yes	1747 (36.04)	14,175 (34.82)	
Work length (year)	13.93 ± 8.97	13.30 ± 9.48	*t* = 4.62*
Current duty			
Fire suppression	1968 (40.40)	17,028 (41.71)	*χ2* = 28.68*
EMS	1468 (30.14)	13,203 (32.34)	
Officer	1435 (29.46)	10,596 (25.95)	
Recent trauma			
No	1094 (22.46)	16.34 (39.27)	*χ2* = 525.01*
Yes	3777 (77.54)	24,793 (60.73)	
Occupational stress	7.94 ± 1.62	7.07 ± 1.65	*t* = 35.06*
Emotional labor	16.11 ± 4.66	12.58 ± 5.04	*t* = 49.50*
Probable PTSD			
No	4240 (87.05)	40,256 (98.60)	*χ2* = 2268.95*
Yes	631 (12.95)	571 (1.40)	
Probable Depression			
No	4454 (91.44)	40,683 (99.65)	*χ2* = 2418.02*
Yes	417 (8.56)	144 (0.35)	

*EMS* Emergency Medical Services; * *p* <  0.001

**Table 2:** Demographic, occupational, and clinical correlates of suicidal ideation in the past year (*N* = 45,555)

	B	SE	OR	95% CI	*p*
Age	0.007	0.002	1.007	1.003–1.012	0.002
Sex					
Male (reference)					
Female	0.394	0.056	1.484	1.328–1.657	< 0.001
Marital status					
Married (reference)					
Never married	0.000	0.049	1.000	0.909–1.101	0.997
Divorced/separated/widowed	0.545	0.095	1.724	1.432–2.076	< 0.001
Religion					
No (reference)					
Yes	0.001	0.034	0.999	0.935–1.068	0.984
Current duty					
Fire suppression (reference)					
EMS	0.271	0.043	0.762	0.701–0.829	< 0.001
Officer	0.398	0.044	1.488	1.366–1.622	< 0.001
Recent traumatic experience					
No (reference)					
Yes	0.614	0.040	1.847	1.709–1.997	< 0.001
Occupational stress	0.175	0.012	1.191	1.164–1.219	< 0.001
Emotional labor	0.091	0.004	1.095	1.087–1.103	< 0.001
Probable PTSD					
No (reference)					
Yes	1.388	0.069	4.008	3.499–4.591	< 0.001
Probable depression					
No (reference)					
Yes	2.188	0.109	8.916	7.201–11.039	< 0.001

*EMS* Emergency Medical Services, *B* regression coefficients, *SE* Standard error of regression coefficient, *OR* Odd Ratio, *CI* Confidence Interval

## Discussion

This is, to the best of our knowledge, the first nationwide epidemiological study investigating the 1-year prevalence of suicidal ideation in firefighters. In this study, we found that 10.66% of Korean firefighters reported having suicidal ideation in the past year, which is higher than in the Korean general population. A nationwide study conducted by the Korean Epidemiologic Catchment Area Study Replication (KECA-R) in 2016 showed that the 1-year prevalence of suicidal ideation in the Korean general population was 2.9%, using the Korean version of the Composite International Diagnostic Interview [46]. From this, we may suppose that more firefighters experience suicidal ideation during their active duty period than the general population with other occupation, indicating that there might be risk factors unique to the occupation of firefighting.

A previous study reported a career prevalence of suicidal behavior among American firefighters [13]; it reported that the career prevalence of suicidal ideation, plan, and attempt was 46.8, 19.2, and 15.5%, respectively. These figures are much higher compared with our results. This difference might be attributable to several reasons. First, their data was obtained from a relatively small sample (1027 firefighters), while our data was obtained from a relatively large sample size (45,698 firefighters). Second, their study included an integrated population of firefighters, ranging from volunteers to retired firefighters, and showed that volunteers were more likely to report suicidal behaviors than full-timers. However, in our study, only full-time firefighters currently working as professionals were analyzed. Third, their study included firefighters with various races and ethnicities residing in the US, while in our study, only Korean – one race and ethnicity – firefighters were enrolled. Stanley et al. recruited White, Hispanic, Latino, Native American and Alaska Native firefighters; and being Native American or Alaska Native was a key factor associated with increased risk for suicidal behaviors. Finally, we analyzed firefighters’ suicidal ideation only in the past year, while Stanley et al. analyzed suicidal ideation in whole career years of firefighters.

In this study, we also investigated the correlates of suicidal ideation in the past year among firefighters. Results showed that PTSD and depressive symptoms were factors most strongly correlated with suicidal ideation in the previous year among demographic, occupational, and clinical characteristics. It is consistent with the previous findings that PTSD and depression were linked to suicidal behaviors [14, 15, 47–50]. Notable finding of this study was that occupational factors were associated with suicidal ideation in firefighters, even after controlling for the effect of PTSD and depressive symptoms. First, we found that recent exposure to traumatic event significantly heightened the possibility of developing suicidal ideation in the past year even after adjusting for demographics as well as PTSD and depressive symptoms. In other words, firefighters who experienced a recent traumatic event could be at higher risk for developing suicidal ideation, despite the lack of PTSD or depressive symptoms, immediately following the traumatic event. This finding is consistent with previous studies, showing that the association between traumatic experience and suicidal behaviors held irrespective of a presence of PTSD [51–53]. It is clear that mental disorders, such as PTSD and depression, accounted for a significant portion of the association between suicidal ideation and traumatic experience. However, these findings suggest that the association between suicidal ideation and traumatic experience does not occur only in the presence of psychiatric disorders and that psychiatric disorders might partially mediate the association. Further study is needed to investigate the interactions between traumatic experience and mental disorders in predicting suicidal ideation.

Second, we found a significant association of occupational stress from physical work condition with suicidal ideation in firefighters. There is accumulated evidence that job-related stress is linked to mental health problems in emergency workers as well as the general population [54–57]. In particular, a previous study conducted in four countries – Korea, China, Australia, and Germany – reported that occupational stress, such as job strain, organizational injustice, and effort-reward-imbalance was associated with suicidal ideation [20]. Recently, the association between suicidality and occupational stress, such as discrimination, inadequate pay, disruption of sleep, and concern about serious injury, was reported among firefighters [19]. However, it has not yet been investigated whether occupational stress from a difficult physical environment of workplace is associated with suicidal ideation in workers. Our findings provide some evidence that working in an unsafe environment or suffering from physical danger on duty could heighten the possibility of suicidal ideation in firefighters.

Third, emotional labor as an occupational stress factor had a positive association with suicidal ideation in Korean firefighters. Firefighters should keep their true feelings in hiding when faced with sickness, death, and violent accidents. Moreover, Korean firefighters usually suffer from emotional labor due to unreasonable demands from the aggressive or picky civil petitioners, presenting similar experiences with those in customer-service industry [24]. A recent study showed that emotional labor made firefighters vulnerable to mental problem by modulating the effects of traumatic experiences on PTSD symptoms [29]. Based on previous findings, it is possible that the high demand of emotional labor might be a risk factor for suicidal behaviors alone and by exaggerating the severity of PTSD.

The final occupational factor related to suicidal behaviors was the officer as a present job position among firefighters. Officer positions include administrators, special investigators, and communicational and informational system operators. Though firefighters in officer position may have less chance to be exposed to traumatic experience during work, they may get highly stressed by high administration work load and pressure, less peer support, and lower salary (e.g. lower danger pay) compared with those in fire suppression or EMS.

Among the demographic characteristics, we found that female gender was significantly associated with the risk of suicidal ideation in the past year. This finding is consistent with the previous report that the 1-year prevalence of suicidal ideation was greater in females than men [58]. Compared with other OECD countries, there are major gender gaps in earning, labor market participation, and representation in the government of Korea [59]. This gender discrimination in Korean society could partly explain the higher risk of suicidal ideation in female firefighters. Given that the occupation of firefighting remains heavily male dominated, female firefighters may feel relatively more discomfort in the occupational environment.

This study has several limitations. First, the cross-sectional design of the study limits its ability to confirm a causal relationship between suicidal ideation and the demographic, occupational, and clinical factors in firefighters. In the future, longitudinal studies should be conducted to confirm the causal relationship found in this study. The current study collected data from a web-based self-reported questionnaire. Self-report assessment has a wide range of tendencies for the participants to respond inaccurately to questions and the recall bias could have possibly influenced the results. Standardized interviews would provide a more accurate and detailed information regarding the prevalence and correlates of suicidal ideation in the population of firefighters. Furthermore, we used a single-item question to assess past-year suicidal ideation and thus, we did not measure a broad spectrum of current and previous suicidal thoughts. Further studies are necessary to investigate the suicidal risk and its correlates using a comprehensive assessment of attitudes and behaviors related to suicide in firefighters.

## Conclusion

The 1-year prevalence of suicidal ideation was higher in firefighters than in the general population. Female gender, divorced/separated/widowed marital status, current duty of an officer, recent traumatic experience, and high level of occupational stress and emotional labor were significant correlates of suicidal ideation in the past year, even after controlling for the effects of PTSD and depression. These findings suggest that consideration and early detection of these correlates may be important in protecting firefighters from the risk of suicide. Longitudinal studies are needed to determine the causal relationships among these correlates, suicidal ideation, and completed suicide.

## Supplementary information

**Additional file 1: Table S1.** The list of traumatic events

**Additional file 2: Table S2.** The list of items on the Korean Occupational Stress Scale and the Korean Emotional Labor Scale

## Data Availability

The datasets used and/or analyzed during the current study are available from the Fire Policy Division in Health Management Team from National Fire Agency of Korea on reasonable request.

## Abbreviations

CI: Confidence interval
DSM-5: Diagnostic and Statistical Manual of Mental Disorders, 5th edition
EMS: Emergency medical services
KECA-R: Korean Epidemiologic Catchment Area Study Replication
KELS: Korean Emotional Labor Scale
KOSS: Korean Occupational Stress Scale
OR: Odds ratio
PCL-5: PTSD Checklist-for the fifth edition of the Diagnostic and Statistical Manual of Mental Disorders
PHQ-9: Patient Health Quetionnaire-9
PTSD: Posttraumatic stress disorder
SBQ-R: Suicidal Behavior Questionnaire-Revised
