# Supplementary material for: Prevalence and correlates of suicidal ideation in Korean firefighters: a nationwide study
Source: BMC Psychiatry. 2019 Dec 30;19:428. doi: 10.1186/s12888-019-2388-9 (PMC6937629; doi:10.1186/s12888-019-2388-9)
Supplement: Supplementary file 1 — Additional file 1: Table S1. The list of traumatic events [file 12888_2019_2388_MOESM1_ESM.docx]

Table S1. The list of traumatic events

| ITEMS |
| --- |
| 1. Witness duty- related death or suicide of co-worker^*^ |
| 1. Co-worker death or suicide (not witnessed)^*^ |
| 1. Experience career ending injury (self) |
| 1. Render aid to seriously injured friend/relative |
| 1. Sudden infant death incident |
| 1. Exposure to hazardous chemicals |
| 1. Severe injury to co-worker |
| 1. Render aid to seriously injured child |
| 1. Fire incident with multiple deaths |
| 1. Multiple casualty motor vehicle accident ( >5 deaths) |
| 1. Third degree burn (self) |
| 1. Multiple casualty motor vehicle accident (1-4 deaths) |
| 1. Fire incident with multiple burn victims |
| 1. Render aid to seriously injured adolescent |
| 1. Render aid to dangerous psychiatric patient |
| 1. CPR/full arrest – family present |
| 1. Render aid to mutilated adult/attempted homicide |
| 1. Treat injured patient who resembles self/spouse |
| 1. Attempted domestic homicide victim |
| 1. Experience head injury (self) |
| 1. Remove the body of a suicide victim |
| 1. Remove a severely decayed corpse |
| 1. Involved in a safety accident that received public spotlight |

*:item 1 regards that direct experience of the death of a co-worker, item 2 regards the indirect experience of the death of a co-worker
