# Supplementary material for: Prevalence and correlates of suicidal ideation in Korean firefighters: a nationwide study
Source: BMC Psychiatry. 2019 Dec 30;19:428. doi: 10.1186/s12888-019-2388-9 (PMC6937629; doi:10.1186/s12888-019-2388-9)
Supplement: Supplementary file 2 — Additional file 2: Table S2. The list of items on the Korean Occupational Stress Scale and the Korean Emotional Labor Scale [file 12888_2019_2388_MOESM2_ESM.docx]

Table S2. The list of items on the Korean Occupational Stress Scale and the Korean Emotional Labor Scale

| ITEMS |
| --- |
| KOSS: Difficult Physical Environment |
| 1. My workplace is clean and comfortable |
| 1. I am exposed to dangerous work and possibility of high risk of accidents |
| 1. I have to work for a long time taking uncomfortable posture |
| KELS: Emotional Damage |
| 1. My pride is often hurt when dealing with people |
| 1. I am hurt when I have to hide my real emotions |
| 1. I feel my emotion as any commodity when dealing with people |
| 1. Hard feelings remains even after work |
| 1. I am very hurt during the process of dealing with people |
| 1. It is emotionally difficult as I have to do my best dealing with people even when I am very tired |

KOSS: Korean Occupational Stress Scale; KELS: Korean Emotional Labor Scale
